# An Essay on the Pathology and Therapeutics of Scarlet Fever

**Published:** 1858-11

**Authors:** 


Art. II.—*An Essay on the Pathology and Therapeutics of Scarlet Fever.* By Caspar Morris, M.D., Fellow of the College of Physicians of Philadelphia, Member of the American Philosophical Society, etc. Second edition. 8vo., pp. 189. Lindsay & Blakiston: Philadelphia, 1858.

To every one sufficiently familiar with scarlatina to have felt how imperfect is our knowledge of its pathology, and how powerless our resources for the relief of some of its severer forms, the announcement of a work devoted especially to its consideration, and embracing in its pages "the result of more than thirty years' observation in various public institutions as well as private practice," will be received with pleasure; in the hope that the teachings of so long an experience may have revealed to the author clearer views of its nature, and established the principles of a more successful treatment. The volume that comes before us thus recommended, is essentially the republication of a course of lectures which appeared in the *Medical Examiner* for 1851, although very much enlarged and improved, by the addition of new cases, and more recent observations, together with an alteration in the style of the work throughout, adapting it to its new position as an essay. It is no doubt due to its original lecture form, that we find in the treatise a want of strict method, that interferes somewhat with the convenience of ascertaining the author's views upon particular points without overlooking the whole book; this would not be felt, however, by those seeking for facts rather than opinions.

After picturing the dread which justly invests scarlet fever, from its treacherous nature and the rapidity of its course, the author proceeds to the "careful investigation of its character, cause, and treatment." For convenience of description he arranges the cases, according to the usual division, into simple, anginose, and malignant, designating as irregular, "those which are not reducible within the limits of either of these classes, though, evidently, derived from the same origin." He reminds us, however, "that divisions are arbitrary," and that "all varieties are but phases of one disease, which fall into each other by imperceptible shades, so that while the cases which may be assumed as types of each class have strong marks of distinction, there are others which it is difficult to distribute to either." The simple form he assumes as the normal condition of the disease, being that which is generally found in sporadic cases,—although sometimes prevailing in wide-spread epidemics,—while the anginose and malignant, associated with irregular types, predominate during every severe epidemic prevalence of the disease. The descriptions that follow these different forms are clear and full, consuming upwards of twenty pages, and including many curious illustrative cases, especially in the malignant and irregular divisions. In speaking of important symptoms the author lays much stress upon the frequent pulse. He says: "the physician is sent for, and finds the pulse beating with a rapidity almost pathognomonic of the disease, so that if precluded from any other mode of investigation, he might, with but little danger of error, decide the case to be one of scarlet fever. There is no disease in which there is the same condition of the circulation, with the single exception of the fever of puerperal women." This acceleration of the circulation in scarlet fever seems to have been constantly observed, and by all acknowledged to exceed that of febrile diseases generally of the same degree of violence. It expresses irritability rather than inflammation, and is probably due to the impression of the poison on the sympathetic nervous system, or directly on the muscular tissue of the heart and vessels. It varies with different epidemics and forms of the disease, and is increased by any measure that reduces the vital force of the patient. Extreme frequency of pulse, without proportionate strength, therefore, must be regarded as an early indication for supportive treatment. The author notices greater frequency of pulse, with less force, in the anginose than in the simple form, "corresponding in this respect with the general want of tone of the whole system." Of symptoms referable to the skin the author alludes to the heat of surface, the eruption, and desquamation. He considers "it has been a common error to suppose that the more vivid the eruption the more favorable was the prognosis. Enlarged experience has disproved to me this assumption; many fatal cases are marked by a rash of an intensely bright color and universally diffused." This last assertion is undoubtedly so, but does not, therefore, destroy the more general truth of common opinion upon this point. Vividness of eruption indicates a corresponding integrity of vital force, and although such cases are liable to fatal issue, the mortality among them is not so great as among those asthenic forms characterized by dull and deficient efflorescence.

Of desquamation, he speaks, as "one of the most uniform and characteristic features of the disease," by some writers regarded as a constant concomitant, occurring even in those cases where there is no unusual heat of skin nor eruption. This process, usually considered due to disturbance of nutrition, resulting from congestion of the cutaneous capillaries, is, by Dr. Graves, as cited by the author, ascribed "to a specific influence of the peculiar miasm, rather than to an inflammation of the dermoid tis sue itself;" an explanation, however, that does not throw much light upon the special pathology of exuviation.

In relation to the phenomena of disease manifested in the mouth and throat, the author remarks: "The eruption of scarlet fever is *always* found in the throat, if it affect the external surface; I do not mean the inflammation which constitutes the *anginose* variety. Even in the most simple case the mucous membrane presents an appearance quite analogous to that of the skin. The projection of the elongated papillæ through the white fur on the tongue, once thought pathognomonic, is less to be relied on. I have been deceived by it. Not so, however, the appearance of the tongue on the second or third day. The sudden throwing off of the coat of fur, while the surface is left red and glossy, is decidedly characteristic, and will never deceive, as it occurs in no other disease at this early period."

The tendency to diffuse inflammation, followed by exudation of lymph, with or without ulceration of the tissue beneath, is particularly noticed on account of its liability to extend to adjacent parts, and thereby give rise to serious complications, The author states that "in the anginose malignant forms, the extension of the disease to the larynx and trachea is by no means uncommon," and he believes more careful observation will confirm the suspicion he entertains, "that the disease known as diphtheritic croup—which begins with inflammation of the tonsils and exudation of lymph, gradually extending to the larynx—is one of the forms of disease now under consideration." He is led to this conclusion by having observed that such cases are particularly prevalent at the times when the scarlet fever influence is most active, and from their evident dependence on some other cause than the mere vicissitudes of temperature, which give rise to the inflammatory croup. The rapidity of the circulation, the tendency to gangrene of the throat, the general similarity of all the symptoms to those cases of undoubted scarlet fever, in which the disease extends to the larynx, the frequent appearance of several cases in immediate succession in a family, and its occurrence simultaneously with the prevalence of malignant scarlet fever, all tend to confirm the author in this impression.

The formation of false membranes is not more frequent in scarlet fever than in the other exanthematæ; it is associated with catarrh, with hooping-cough, and in fact generally with those diseases liable to affect the mucous membrane of the lungs or throat, when occurring in a severe epidemic form; this diversity indicates that diphtheritic exudation is not peculiar to any disease, but that it depends upon a morbid condition of the blood, which may be brought about by a variety of circumstances, and frequently is by those that favor the development of epidemics.

The term diphtheritic croup is in general received as synonymous with the pseudo-membranous form; but when our author speaks of it as beginning with inflammation of the tonsils, of its tendency to gangrene of the throat, and of its disposition to assume epidemic features, he evidently does not refer to primary pseudo-membranous croup, but to that secondary form which follows pseudo-membranous inflammation of the fauces, and which more properly should be considered as a complication of that disease. If this be our author's meaning, he is not the first to entertain a suspicion of its identity with scarlet fever. Dr. Wood, in his *Practice of Medicine,* says: "From its resemblance to the malignant sore-throat of Fothergill, Huxham, and others, with which by some writers it is maintained to be identical, and from the simultaneous occurrence of both these affections with epidemic scarlatina, and the close analogy of their local symptoms with the throat affection of that disease, the inference is not without plausibility, that they may all be produced by the same cause, in those cases in which they have an epidemic or contagious character." There are many reasons, however, that oppose the idea of identity, even between diphtheritic angina and scarlet fever. A prominent one is the fact, that the diseases are not mutually protective of each other, which should be the case if they were of one origin. No disease is less liable to secondary attacks than scarlatina, no matter in which of its varieties it may occur; and yet, it is not uncommon for membranous angina to occur in persons previously affected with scarlet fever. The following illustrative paragraph is from the *Dictionary of Practical Medicine,* by Dr. Copland. He says: "Some years since I attended, early in the winter, some of the children of a numerous family, residing a few miles from town, in a low and damp situation; they had had scarlatina, with very severe sore-throat, two or three years previously. On this occasion one of the oldest was seized with malignant angina, extending to the pharynx, and along the eustachian tube to the ear, with fœtid respiration and irritation of the larynx, producing a constant tickling cough. A similar affection spread to four of the younger children, and in two of them it was complicated with croup; the symptoms of which were severe, continued, and well marked in one, and more spasmodic and intermittent in the other. In these, ash-colored exudations covered the greater part of the fauces and tonsils, and extended down into the pharynx."

The occurrence of pseudo-membranous croup as a *complication* of scarlet fever, especially of its anginose form, is not uncommon, although as a rule in this disease, inflammation falls upon the digestive mucous membrane rather than upon that of the air-passages.

Dr. Tweedie states: "That in the dissections of scarlatina with anginose inflammation, which he has made, he has not seen an instance of membranous exudation extending into the larynx." On the other hand Dr. Copland says: "When the angina attending scarlatina is not of a malignant kind, the larynx and epiglottis seldom betray disorder. But in malignant cases, and in adults, especially those who have been addicted to the use of spirituous liquors, or whose constitutions are broken down, this extension of inflammation to the larynx and trachea is not rare."

Other numerous complications are observed and described, but do not call for particular mention. In relation to the diagnosis of scarlet fever there are many points of interest. In those cases where the sequence of morbid phenomena is uninterrupted throughout, they are in themselves quite sufficient to distinguish the disease; but in scarlet fever, as in other diseases produced by contagion, it often occurs that the whole train of symptoms which usually characterize the malady are not present—one or more being suppressed, or so faint as to escape observation. Under these circumstances, we are compelled to rely upon external conditions, exposure to infection, or the existence of an epidemic to indicate the character of the disease. Happily in practice, sporadic cases, that might not otherwise be suspected of originating in a specific cause, are apt to be stamped with all the ordinary features of the fever,—the eruption, frequent pulse, hot skin, etc., while those of greatest doubt occur during epidemics, when the attention is fully aroused to the prevalence of the malady. Unquestionably, coincident existence of the disease justly decides the origin of many a perplexing case; this is particularly felt in those irregular types, where death occurs so early as to give no time for the development of consecutive symptoms, and where the diagnosis must necessarily be, in a great measure, inferential.

The presence of the eruption was at one time considered essential to establish the existence of true scarlet fever. But the occurrence in individuals of a constitutional affection of similar duration to scarlet fever, having the rapid pulse, the diseased throat and fauces, desquamation of the cuticle and consecutive dropsy, at the same time the capability of propagating eruptive forms of the fever, has been so fully and frequently attested, that the unity of origin of the two conditions is now scarcely doubted, and by some authors this peculiarity has been adopted as the basis of a new division. But further than this, there are cases in which there is not only absence of the eruption, but also of the sore-throat. These are mentioned by Dr. Copland as latent scarlatina, and considered by him to be due to the same cause as the ordinary forms, from their occurrence in different individuals of the same family at the same time, from having similar complications, and more especially from "the very frequent appearance, in these cases, of renal affection, of albuminous urine, and consecutive dropsy or inflammation."

Our author adds his testimony to the frequent occurrence of such cases in certain epidemics, and speaks of them as presenting "merely febrile disturbance, followed by desquamation and anasarca." From these considerations it appears that fever, with unusual frequency of pulse, an eruption on the second day, with anginose symptoms, and after that desquamation of the cuticle and subsequent dropsy, constitute typical scarlet fever, and that each or many of these symptoms may be partially or entirely suppressed, leaving others in position to characterize the disease. Dr. Morris dwells at some length upon the means of distinguishing this disease from measles, variola, and typhus fever, and mentions a rash often accompanying indigestion in children, that very much resembles it, but that may be recognized by the less frequent pulse, the subsequent absence of desquamation, and the condition of the tongue on the second or third day in scarlet fever, which is left red and glossy by the sudden throwing off of the coat of fur.

Concerning the cause of scarlet fever our author does not long delay. He says: "That it depends on some unknown agency, producing what is called epidemic influence, no one denies. In what this influence consists it is impossible to determine with our present limited knowledge and means of observation."

In proof that this "unknown agency" is a specific material cause, Dr. Carpenter thus reasons: "It may be admitted that our belief in a specific material cause for a great part of the effects set down to the action of 'morbid poisons,' is merely inferential. But it must be remembered that the evidence of chemistry itself is often purely inferential, for we recognize the presence of a chemical substance, not merely by obtaining it in a separate form, but by witnessing the reactions which it displays with various tests; and there is one substance, fluorine, which has never yet been isolated, and of whose existence, however, no chemist would hint a doubt. Even could we obtain these poisons in a separate state, and could subject them to chemical analysis, we should know much less of their most important properties than that which we can ascertain by observations of their actions in the system; this alone affording the means of judging of their dynamical character, which is of far more importance than a knowledge of their chemical composition. In the case of those poisons capable of being introduced by inoculation, we have indeed the required proof of their material existence, and this proof is capable of being extended, by a safe analogy, to infectious diseases generally. For if smallpox can be communicated by the inhalation of an atmosphere tainted with the exhalations of a person already affected with it, as well as by the introduction of the fluid of the cutaneous pustule into the blood of another, it can scarcely admit of a question, that the same poisonous agent is transmitted in both cases, although through different media, and that it has as real an existence in the transferred air as in the transferred pus."

Efforts have been made to obtain, by inoculation equally, strong evidence of the material character of the poison of scarlet fever that exists in smallpox, but with varying success. Dr. Copland, however, considers it has been demonstrated. He states that Sir B. Harwood and others have tried to inoculate healthy children with the fluid from vesicles, sometimes intermingled with the eruption of scarlatina, in hopes of producing a milder disease, as in smallpox; but although the disease was thus communicated in many instances, no mitigation of its type was thus obtained. In a case which came under his own care, the disease was produced by the contact of a small portion of the discharge from the throat of a person with malignant anginose scarlatina, and the patient thus infected had the disease in the most severe form, and recovered with difficulty. The conclusiveness of these cases is impaired by their seeming to indicate that different forms of the disease have the property of reproducing their own types when inoculated, a fact at variance with the experience of inoculation in smallpox, and conflicting with that afforded by the well-known diversity of cases occurring during every epidemic prevalence of disease. Our author advocates warmly the contagious nature of scarlet fever, regarding the question as one not merely of speculative interest, but possessing highly important practical bearings. He believes the principle of contagion may attach itself to the clothing worn by patients, or long exposed to it, and thus transmit the disease, but his own observation leads him to deny the possibility of communication by mere transient intercourse. He cites many curious and interesting cases corroborating these views. The period of incubation he considers more uncertain in this disease than in any other of the same class. The extreme lengths of the interval between exposure and sickening, that have come to his knowledge, are forty-eight hours, and twenty-eight days; from eight to fourteen days being, however, the usual interval.

In speaking of epidemics of scarlet fever, Dr. Morris notices their tendency to occur at irregular periods, the intervals being marked by the prevalence of the disease in a mild form, or its entire disappearance; for these intervals are at times so protracted that a recurring malignant epidemic has repeatedly been described as a new disease, a whole generation of medical practitioners passing away without its appearance. He mentions that in the year 1831 a highly respectable physician, very extensively engaged in practice, told him he had never seen a fatal case of scarlet fever, his testimony corresponding with that of the bills of mortality for the period during which he had pursued his professional career. In some instances a grand epidemic cycle appears, composed of series of minor epidemics.

Our author thinks secondary attacks of scarlet fever do occur, although less frequently than in some of the other exanthematous fevers; and that, as in variola and measles, they are more frequent in those who have suffered with its most severe forms. It would appear as though, in those persons, there was a greater degree of susceptibility to the influence of the contagious principle or epidemic miasm, while there are others in whom this liability exists but slightly at any period of life. As a rule, however, one attack of the malady gives to the person immunity for life. The question, on what does this susceptibility depend? is not settled, some authorities referring it to changes in the blood, others to altered states of the organic nervous system. Dr. Carpenter, after speaking of the tendency to increased susceptibility during depressed conditions of the vital powers of the system, when there is a tendency to putrescence in the excreted matters, as in the puerperal state, or when there is a special source of decomposing matter in the body, says: "Thus then we may affirm with strong confidence, that the liability to zymotic disease depends upon a previous condition of the blood, and more especially on the presence of fermentable matters resulting from the ordinary processes of disintegration, which, in the state of perfect health, are eliminated as fast as they are formed, but of which an accumulation is prone to take place, either when there are special sources of an augmented production, or when the excretory operations are imperfectly performed."

On the other hand, Dr. Copland says: "That the immunity obtained by an attack of those diseases which infect the constitution only once cannot be imputed to any change in the blood consequent to such infection, may be inferred, first, from the impossibility of a permanent change in this fluid, that could prevent the recurrence of any alteration in it which had taken place on some former occasion; and secondly, from the gradual and entire renewal of this fluid, after longer or shorter periods, a renewal of susceptibility inevitably supervening, if this property resided in the blood. We must therefore refer the immunity from a second infection to the organic nervous system, and view the susceptibility of this system to have been so affected or specifically changed by the first operation of the poison as no longer to be capable of being roused, by any subsequent application of the same species of poison as previously affected it, to a similar series of morbid changes and actions."

"We may now proceed," with the author, "to the consideration of certain results of scarlet fever, which are more durable than the disease itself, quite distinct in their character, equally, perhaps more, to be deprecated than the primary disease, known as sequelæ." He divides them into two classes:—

First. "Those which appear to be a mere continuation of the local lesions developed during the course of the disease and confined to the same vicinity: sloughing and ulceration of the throat; diseases of the ear, often extending to the bony structure; abscesses about the neck; and, in some rare cases, disease of the cervical or dorsal vertebræ, resulting in caries;" and—

Second. "Those which are less directly connected with primary causes and congestion of the kidneys." Among the most important of the second class, the author places "that destruction of the cuticle by the cutaneous inflammation, which gives rise to the process of desquamation." And considers, at length, the question, how far other sequelæ, inflammation of the kidneys, dropsical effusion, and endocarditis, may depend upon this interruption of the functions of the skin.

Disease of the kidney he does not attribute to this cause, but supposes both organs affected simultaneously by one agency; that the same diseased action may be established in the Malpighian bodies and epithelium of the uriniferous tubules, as is observed on the external integument; this he thinks probable from the fact that the flow of urine is always much diminished about the time at which desquamation is at its height, and by the appearance, at this juncture, of epithelial scales, abundantly in the urine. He considers both the skin and kidney are equally interested in the production of sequelæ, accompanied with serous effusion. These he considers in the order of frequency in which they occur; first the cellular tissue of the skin, then the pleural sac, or the pulmonary tissue, and after this the ventricles of the brain and the pericardium. Cerebral effusion he regards as the most formidable of this group. It is indicated by the occurrence of stupor, or violent headache and vomiting; the pulse is slow; one or both pupils dilated; vision is impaired, or lost entirely; and there may be strabismus, or even general convulsions, or paralysis. A doughy, pallid, puffed face, is always to be considered an indication of danger, even though the quantity of urine is large. The urine must not only be abundant, but charged with its saline constituents. It is not the mere leakage of watery fluid that restores the normal condition of the blood, and until this is effected, the patient is still liable to serious complications. There is much discrepancy concerning the pathology of this condition; it is ascribed by some to uræmic poisoning of the blood, by some to meningitis, and by others to mere passive effusion of serum into the meshes of the pia-mater, the ventricles of the brain, or into the substance of that organ. Our author attributes it to the combined influence of a poisoned state of the blood—partly due to the primary cause of scarlet fever, partly to the impaired action of the skin, and still more to the interruption of the function of the kidneys caused by the congestion, and, perhaps, inflammation of these organs—and serous effusion within the cranium; nor is he at present disposed to attribute the effusion to active inflammation of the encephalon.

Before entering upon the details of treatment, the author remarks: "That there is one idea which should be ever present to the mind, as the regulating principle by which every plan of treatment must be conducted, and which shall place decided limits to the extent to which any course may be followed. Whatever peculiarities may mark the epidemic character, or vary the individual case, scarlet fever, in all its varieties, is a disease which has a specific origin, a fixed course, and a certain termination." The effort on the part of the physician should be, therefore, not to curtail the duration of the disease, but to assist the patient to endure it, and avert, if possible, tendencies to local destruction.

In the simple form, the treatment suggested consists of a mild laxative of calcined magnesia, given on the first day, followed by a gentle refrigerant, as lemonade, with neutral mixture, and a small quantity of spirit of nitrous ether, to promote diaphoresis; the avoidance of fatigue and exposure, and the adoption of a careful diet, not only during the four days of febrile action, but until the desquamation is complete. No animal matter, either solid or in the form of broth or soup, should, on any account, be allowed during the first four days. When the nervous symptoms are more marked with twitching, trembling, or delirium, cutaneous ablutions should be employed conjointly with the former treatment; tepid sponging, or affusions of water at 96° or 98° of Fahrenheit, will be proper forms when the skin is hot and dry, the pulse quick and strong, with restlessness and delirium; when there is violent delirium, with great heat of skin, very frequent pulse, convulsions, or at least violent muscular twitchings, the cold douche to the head, maintaining the impression by cloths wrung out of ice-water, while the body is sponged with water at the temperature of the room, will be found the most efficient means to procure tranquillity. Bleeding, emetics, and the more violent therapeutic measures, the author disapproves of, preferring to rely on simple means, with entire rest. In the anginose form the only change of treatment usually demanded is external warmth and moisture, with the free use of ice internally; as the primary fever subsides, if the pulse loses its frequency, the skin becomes cool and moist, and the patient languid, quinine, given in moderate doses, at intervals of two or four hours, is highly important. Where the eruption is imperfectly developed or receding, capsicum is productive of the happiest results. It may be given, in weak broth, with safety, even to the youngest infants. In the malignant form the author recommends an emetic of ipecacuanha, or of eupatorium perfoliatum, early in the treatment; giving capsicum subsequently, to support vital power and to arrest local lesions in the throat; chlorate of potash may be given as an alterative, and when stimulation is necessary, wine-whey, beef-broth, highly seasoned with capsicum and quinine. The stimulating plan of treatment here advocated may prove pernicious if employed before there is unmistakable evidence of the failure of vital power. This, therefore, should carefully be regarded.

After considering at length the possibility of removing the susceptibility to scarlet fever by belladonna, the author thus concludes: "Though I have thus entered at large into the question on account of the positive assertions of the advocates of prophylactic treatment, and have endeavored to exhibit the manner in which, if at all useful, it operates, I have no faith in the prophylactic power of this or any other agent."

In the treatment of sequelæ Dr. Morris does not hesitate to use active antiphlogistic measures; they are inflammatory in character; and in severe cases affecting vital organs, the safety of the patient depends upon prompt and vigorous depletion. Local and general bleeding, calomel, digitalis, aconite and nitrate of potash, are some of the means he advises for their relief.

